# Perspectives on computational modeling of biological systems and the significance of the SysMod community

**DOI:** 10.1093/bioadv/vbae090

**Published:** 2024-06-26

**Authors:** Bhanwar Lal Puniya, Meghna Verma, Chiara Damiani, Shaimaa Bakr, Andreas Dräger

**Affiliations:** Department of Biochemistry, University of Nebraska-Lincoln, Lincoln, NE 68588, United States; Systems Medicine, Clinical Pharmacology and Quantitative Pharmacology, R&D BioPharmaceuticals, AstraZeneca, Gaithersburg, MD 20878, United States; Department of Biotechnology and Biosciences, University of Milano-Bicocca, Milan 20126, Italy; Department of Medicine, Stanford Center for Biomedical Informatics Research (BMIR), Stanford University, Stanford, CA 94305-5479, United States; Computational Systems Biology of Infections and Antimicrobial-Resistant Pathogens, Cluster of Excellence ‘Controlling Microbes to Fight Infections’, Institute for Bioinformatics and Medical Informatics (IBMI), Eberhard Karl University of Tübingen, Tübingen 72076, Germany; German Center for Infection Research (DZIF), partner site Tübingen, Tübingen 72076, Germany; Quantitative Biology Center (QBiC), Eberhard Karl University of Tübingen, Tübingen 72076, Germany; Data Analytics and Bioinformatics, Institute of Computer Science, Martin Luther University Halle-Wittenberg, Halle (Saale) 06120, Germany

## Abstract

**Motivation:**

In recent years, applying computational modeling to systems biology has caused a substantial surge in both discovery and practical applications and a significant shift in our understanding of the complexity inherent in biological systems.

**Results:**

In this perspective article, we briefly overview computational modeling in biology, highlighting recent advancements such as multi-scale modeling due to the omics revolution, single-cell technology, and integration of artificial intelligence and machine learning approaches. We also discuss the primary challenges faced: integration, standardization, model complexity, scalability, and interdisciplinary collaboration. Lastly, we highlight the contribution made by the Computational Modeling of Biological Systems (SysMod) Community of Special Interest (COSI) associated with the International Society of Computational Biology (ISCB) in driving progress within this rapidly evolving field through community engagement (via both in person and virtual meetings, social media interactions), webinars, and conferences.

**Availability and implementation:**

Additional information about SysMod is available at https://sysmod.info.

## 1 Introduction

In the last few decades, the study of biological systems has witnessed a paradigm shift driven by recognizing inherent nonlinearity and the involvement of diverse molecular players. Systems Biology, an interdisciplinary field integrating biology, mathematics, statistics, and computer science, addresses exploring collective behaviours in biological systems that elude traditional molecular approaches. Central to this evolving paradigm is the adoption of mathematical and computational models for simulating complex systems, drawing inspiration from the rich framework of complex systems science borrowed from physics. This modeling field is commonly referred to as Computational Systems Biology. Its scope extends across the molecular, cellular, organ, and tissue levels to the population and ecosystem levels. Advancements in next-generation sequencing and computing have encouraged the use of mathematical and computational techniques to model and simulate biological processes with these complex datasets. These computational models allow scientists to simulate experiments, predict the outcomes of biological processes, and iteratively generate new testable hypotheses.

At the molecular scale, systems models help in studying biochemical processes, cell signalling, protein interactions, and the regulation of genes. Such molecular scale models help us understand how cells work fundamentally facilitating advancements in drug discovery, disease treatment, and biotechnology (Gu *et al.*, 2019, Saez-Rodriguez and Blüthgen, 2020). Computational models can be used to explore cell interactions, including cell-to-cell communication and the cell dynamics in a population of bacterial communities (Meier-Schellersheim *et al.*, 2019, van Vliet *et al.*, 2022). This technique provides insights into the cell groups’ emerging behaviour and collective effects. Further, computational modeling can be applied to population, ecological, and evolutionary contexts (Creanza *et al.*, 2017, Muhammad-Kah *et al.*, 2019, Bentley and Lim, 2022, Cosme *et al.*, 2023). Ecological models help us understand how different species interact, their population dynamics, and the stability of entire ecosystems (Evans, 2012, van den Berg *et al.*, 2022). These shed light on the complex relationships and processes that govern natural environments. Computational models in evolution are used to study biological change and evolutionary trajectories (Eccleston *et al.*, 2023), the adaptation of genes, genetic or phenotypic variation (Almendro *et al.*, 2014), and examine how species adapt to their environments (Xue *et al.*, 2019), and the emergence of new traits over long periods, helping us understand the mechanisms of evolution.

**Figure 1.**
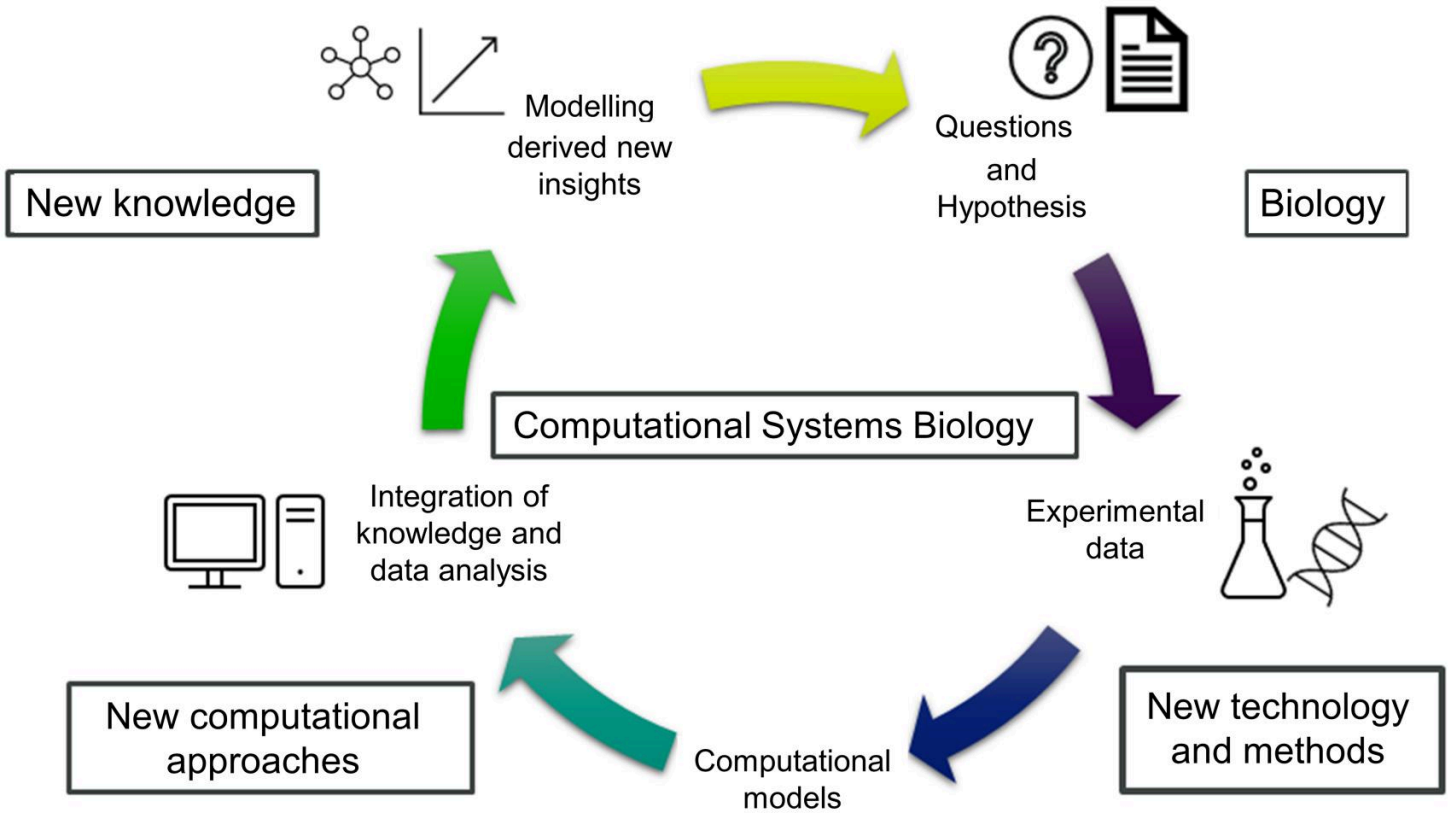
Overview of computational systems biology and modeling. The iterative process used in computational systems biology modeling comprises formulating biological questions and hypotheses, using experimental data to support these, and building computational models. The modeling-derived data analysis helps in knowledge integration and new insights, which help formulate new hypotheses. The figure is inspired by Martins dos Santos *et al.* (2022).

The rapid advancement of cutting-edge technology in molecular biology, such as single-cell technology, resulted in a wealth of genomics, transcriptomics, proteomics, and metabolomics data. Adopting multi-omics approaches that span different molecular layers and combine these data can provide a comprehensive and holistic understanding of complex biological systems (Pinu *et al.*, 2019). Computational models that integrate such diverse data sets employing mathematical, machine learning (ML), and artificial intelligence (AI) approaches are crucial to study complexity of biological processes (Fig. 1) and for more targeted and effective therapeutic interventions, shaping the future of personalized medicine and biotechnology (Alber *et al.*, 2019).

In addition, computational models, such as systems pharmacology models, are important in drug discovery (Bradshaw *et al.*, 2019), aiding pharmaceutical research by predicting drug-target interaction, reducing reliance on trial-and-error methods, and streamlining the drug development process. Some applications include the determination of dosing regimens, patient stratification, understanding the mechanism of action of the drug and disease modeling; these models facilitate the analysis of gene interaction networks, enhancing our understanding of genetic disorders and helping develop gene therapy techniques (Bradshaw *et al.*, 2019, Bai *et al.*, 2020). Computational modeling is integral to the advancement of personalized medicine, where it aids in tailoring treatments to individual genetic profiles, studying treatment efficacy, and identifying adverse effects. These applications of computational modeling in biological systems demonstrate its transformative impact across various life sciences domains.

In the forthcoming discussion, we briefly discuss the latest advancements in systems modeling in biology, the challenges the field currently faces, and the role of the SysMod community in highlighting the influential advances of the systems biology field. Lastly, we highlight the future perspectives and the vision of the SysMod community.

## 2 Recent developments in the computational modeling of biological systems

The progress in computational modeling in systems biology has been remarkable, evolving from simple models focused on small-scale molecular interactions and individual biological processes. Technological advancements in molecular biology and biomedical fields have generated tremendous amounts of data that allowed the evolution from simple to more complex models. With the ever-growing computational power and sophisticated algorithms, biological models can now encompass nearly complete cellular functions and organism physiology. AI with more powerful and efficient methods for analysing and integrating complex biological datasets further transformed the field and expanded the possibilities for modeling and simulation.

A significant breakthrough in this domain is creating whole-cell to multi-scale models representing cells and tissues. The journey began with a model of the unicellular organism *Mycoplasma genitalium*, which included all the processes and interactions and a combination of various mathematical approaches (Karr *et al.*, 2012). Researchers are now progressing toward constructing models representing whole-cell processes for more complex systems, such as humans. These models aim to mimic the functionality of an entire cell, a tissue, an organ, and, potentially, a whole organism. More complex models of a complete system, such as human immune systems, referred to as ‘digital twins’, are being designed with such a quality that they can be employed for computational experiments to predict real-life outcomes, such as disease treatment scenarios (Laubenbacher *et al.*, 2022). The potential applications of these models are vast, ranging from gaining insights into disease mechanisms, studying response to treatment, and improving treatment strategy. By creating a digital twin of a human, it becomes possible to simulate the disease progression and effects of treatments at an individual level leading to more effective and targeted therapies.

Multiscale models are analysed using approaches such as the agent-based modeling. An agent is an entity with specific properties that follows certain rules and interacts with its environment based on the defined rules. This type of modeling refers to the dynamic process of the agent interaction, which is simulated repeatedly over time. Multi-scale models can integrate differential equation-based modeling methods like ordinary differential equations (ODEs), partial differential equations (PDEs), and other methods Boolean, and constraint-based models (CBMs). These multiscale approaches allow for detailed representation of multiple biological scales while also modeling distinct behaviour. These are instrumental in biological systems, where agents can represent anything from molecules to cells or entire organisms interacting in a dynamic environment.

ODEs and PDEs are often used to model continuous processes such as biochemical reactions, and transport and diffusion of molecules. In contrast, Boolean models and CBMs effectively represent regulatory networks and metabolic pathways. On a single scale all of these methods have valuable applications in biotechnology and biomedical fields. An example of a multiscale hybrid model employing agent-based modeling, ODE, and PDE approaches effectively capturing the immune response dynamics during *Helicobacter pylori* colonization of the gastric mucosa was demonstrated in Verma *et al.* (2019). Another multi-scale, multi-approach model was used to study the dynamics of CD4 T cells at molecular, cellular, and systemic scales in response to influenza infection (Wertheim *et al.*, 2021). Further, metabolic pathway modeling has been used in drug discovery against immune-mediated diseases (Puniya *et al.*, 2021, Bessell *et al.*, 2023) and infectious diseases such as COVID-19 (Renz *et al.*, 2021). Researchers can capture biological systems’ discrete and continuous aspects by combining different methods within a multi-scale hybrid agent-based framework, leading to a more comprehensive understanding of these complex systems and diseases.

Stochastic dynamic modeling is another field with a significant advancement in describing and analysing complex systems characterized by randomness. These models integrate probabilistic elements to accommodate biological systems’ intrinsic variability and unpredictability. They are crucial for capturing the dynamic behaviour of biological processes influenced by noise, such as gene expression, cellular signalling, and ecological interactions. One application of stochastic dynamic modeling is to study the transitions of cell states. The cell state transition assessment and regulation (cSTAR) approach utilized omics data and ML to develop mechanistic models of core signalling networks governing cell fate transitions (Rukhlenko *et al.*, 2022). Zhou *et al.* employed a multiscale reduction technique to elucidate the underlying stochastic dynamics dictating cell-fate transitions, focusing on cells with transient properties (Zhou *et al.*, 2021).

Additionally, the dimension reduction approach of landscapes for complex dynamical systems facilitated the mapping of high-dimensional systems onto low-dimensional energy landscapes, preserving essential stability and transition information of higher dimension (Kang and Li, 2021). This method was applied to biological networks, including the epithelial–mesenchymal transition and abnormal metabolism in cancer, to identify stable landscapes and define transition pathways. Furthermore, stochastic dynamical models were used to explore the dynamics in ecological systems, such as those involving plant–pollinator interactions (Lv *et al.*, 2024).

The other door-opening development is the integration of single-cell data into computational models. Single-cell technology, including single-cell RNA sequencing (scRNA-seq), has significantly changed computational modeling since they provided new insights into the complexity and heterogeneity of biological processes across cells. This has enabled dissecting the cellular composition of tissues and facilitating an understanding of cell-to-cell variability, which was impossible with bulk analyses. Integrating computational models with single-cell data allows for a more granular view of biological processes at the cellular level, facilitating the understanding of cellular heterogeneity, differentiation pathways, and cell lineage relationships. The computational models incorporating scRNA-seq data could support mapping cells’ developmental trajectories and identifying novel cell types and states in various tissues.

Trajectory inference has massively benefited from interpreting scRNA-seq data in the light of mathematical models, cast into the formalism of differential equations, describing the temporal evolution of precursor and mature RNA molecules from synthesis to decay. This paradigm, known as RNA velocity analysis (Bergen *et al.*, 2021, Furlan *et al.*, 2021), is based on the genome-wide inference of such models on a population of cells. The resulting kinetic rates are then used to predict the evolution of gene expression levels and, consequently, cells’ future states. This results in the detailed ranking of cells on a pseudotime domain and the reconstruction of their temporal interconnections.

Integration of single-cell data, for example, into CBMs of cell populations (Damiani *et al.*, 2019, Wagner *et al.*, 2021), has provided insights into the cellular heterogeneity of tumours, the tumour microenvironment, and the mechanisms of drug resistance in cancer therapy. Computational models that include single-cell data enhance our understanding of tumour evolution, the dynamics of cancer progression, and the biological processes in cancer (Bakr *et al.*, 2023), thereby informing more effective treatment strategies. Single-cell technologies have also revolutionized our understanding of the immune system’s complexity. Computational models incorporating single-cell data are being used to study the dynamics of immune responses, diversity of immune cell types, and mechanisms of immune regulation, which are increasingly being applied in vaccines and immunotherapy development. Single-cell technologies also facilitate the integration of multi-omics data at the single-cell level, providing the genomics, transcriptomics, proteomics, and metabolomics data of an individual cell. This enables a complete understanding of the molecular mechanisms that may drive cellular processes, impacting biological modeling greatly.

Integration of ML with computational modeling increases our understanding of complex biological systems. This interdisciplinary approach leverages the power of ML algorithms for the analysis, interpretation, and prediction of biological data. ML with ODEs, Boolean models, CBMs, and agent-based models (ABMs) have shown substantial progress in understanding complex biological system dynamics. ML techniques, particularly deep learning, have been used to mine and integrate experimental multi-omics and Genome-Scale Metabolic Model (GSMM)-generated data. This integration has revealed previously unknown mechanisms in a sample-specific manner and identified relevant targets (Zampieri *et al.*, 2019). ML is also being used to improve CBM predictions (Schinn *et al.*, 2021). For multi-scale modeling, ML can serve as a tool for integrating various scales of biological data, from cellular to organ levels. For example, clustering, regression, and classification are used with multi-scale models to identify parameters, generate new hypotheses, or optimize treatments (Alber *et al.*, 2019). ABMs have also been integrated with ML techniques. For instance, an integrated ABM regression model was developed to simulate the immune system, using ABM to represent each cell as an agent and employing regression methods for parameter optimization (Tong *et al.*, 2015). This model described immune responses at the cellular level and overcame limitations in parameter estimation in ABM. ML-based surrogate models have been developed to approximate mechanistic models based on ODEs, stochastic differential equations (SDEs), and PDEs (Gherman *et al.*, 2023). Utilizing ML methods for surrogate modeling in ABM enhanced the robustness of sensitivity analyses while simultaneously reducing CPU time consumption during model calibration and analysis (Angione *et al.*, 2022). These surrogates are particularly useful when predictions are needed in real time or when numerous simulations are required. They also help analyse the uncertainty in the predictions of mechanistic models and understand the relationships between inputs, parameters, and outputs. These advancements indicate that ML is augmenting the capability of computational models in simulating complex systems and enhancing their applicability. ML and computational modeling with simulation are creating synergy toward more accurate and efficient exploration of the dynamics of complex biological systems.

## 3 Consequential challenges in the field

Challenges in computational modeling in systems biology span various aspects, from data acquisition to model calibration and validation. Developing computational models requires input from biological data and scientific knowledge. A key challenge is in the integration of heterogeneous data types, such as genomics, transcriptomics, proteomics, metabolomics, and epigenetics—specifically when the data have been acquired from different studies or at different times. Data quality and completeness are crucial, as datasets often suffer from missing values and biases. Robust batch effect correction methods are needed when cross-layer datasets are integrated for model-building purposes. The processing of massive high-throughput data requires robust computational infrastructures and efficient data processing algorithms. The need for interoperability and standardization is prominent, as it facilitates effective data sharing and collaborative research. As computational models become increasingly used to inform clinical, industrial, and environmental decisions, their ethical and regulatory implications become important. Balancing scientific advancement with ethical considerations is vital.

The inherent complexity of biological systems poses a significant challenge in creating accurate models. With more realistic representation of biological processes at various scales, they become more complex, leading to development, parameterisation, and validation difficulties. Parameterization of the model is an essential yet problematic aspect since many parameters in biological systems are difficult to measure directly, leading to reliance on estimations that can be imprecise due to noisy and incomplete data. In this direction, ML has been found to be useful; recently, neural networks have been employed to estimate enzyme kinetic parameters (Kroll *et al.*, 2021, Li *et al.*, 2022, Yu *et al.*, 2023). These methods used data such as protein sequences and substrate structures to train models to predict parameters like Michaelis constants (Km), enzyme turnover numbers (kcat), and catalytic efficiency. Furthermore, a deep learning framework was developed to reconstruct kinetic models for dynamics study (Choudhury *et al.*, 2022).

Another concern is overfitting, where models are overly tailored to specific datasets that can limit the generalizability and applicability of the models. Model validation is challenging, given the limited scope of experimental data available for comparison, specifically high-throughput perturbation datasets under the matching conditions in which the models were developed. Understanding how uncertainties in data and parameters affect model predictions is paramount. Developing robust sensitivity and uncertainty analysis methods is challenging due to biological data and processes’ inherent variability and complexity. Moreover, model scalability is critical; models must be scalable to accommodate the increasing size and complexity of biological datasets while maintaining computational feasibility. Therefore, defining the model’s scope is crucial to balancing the representation of biological complexities and predictive capabilities.

The simulation and analysis of complex biological models, especially multi-scale, multi-cellular and whole organisms, demand immense computational power. Overcoming these computational constraints involves advancements in computational resources, algorithm development, and data management strategies. In addition, balance between specificity with generalizability, data diversity, and model complexity and scalability are prominent factors that need careful consideration. The absence of standardized protocols and formats for model construction and simulation for complex multi-scale models leads to issues with reproducibility and comparability between studies. Creating generalizable models across different systems or conditions is a substantial challenge. To address the reproducibility issues, multiple ongoing community efforts such as the: (i) Systems Biology Markup Language (SBML) (help establish standard formats), (ii) BioModels (https://www.ebi.ac.uk/biomodels/) (help with manual curation of the models), and the (iii) ‘COmputational Modeling in BIology Network’ (COMBINE) (http://co.mbine.org/) (help annotate and semantically enrich the models) have been instrumental (Malik-Sheriff *et al.*, 2020, Tiwari *et al.*, 2021, König *et al.*, 2023). In addition to these ongoing efforts, ensuring thorough model documentation and consistency (across computational environments) is equally vital for the reproducibility of results.

Interdisciplinary collaboration across biology, computer science, mathematics, and others is challenging because of communication barriers and differences in research methodologies and cultures. However, it is crucial for advancing computational modeling in biological systems. Addressing these challenges requires a concerted effort from the scientific community, involving technological advancements and encouraging collaborative frameworks and interdisciplinary education. The continuous evolution of computational modeling in biological systems depends on overcoming these hurdles, leading to more accurate, reliable, and comprehensive models that can significantly advance our understanding of complex biological processes.

## 4 The SysMod community’s impact on computational modeling

### 4.1 SysMod as a community

In 2016, a community was founded for the Computational Modeling of Biological Systems (SysMod) as a Community of Special Interest (COSI) of the International Society for Computational Biology (ISCB) (Dräger *et al.*, 2021, Niarakis *et al.*, 2022, Puniya and Dräger, 2023). The goal was to bring together members focusing on computational modeling and analysis of biological systems. Since then, SysMod has organized and contributed to the eight annual gatherings at the Intelligent Systems for Molecular BIology, European Conference on Computational Biology (ISMB/ECCB) conferences. SysMod COSI aims to promote the development of novel mathematical tools, present systems modeling approaches, and develop predictive models to enhance the understanding of biological systems.

**Figure 2.**
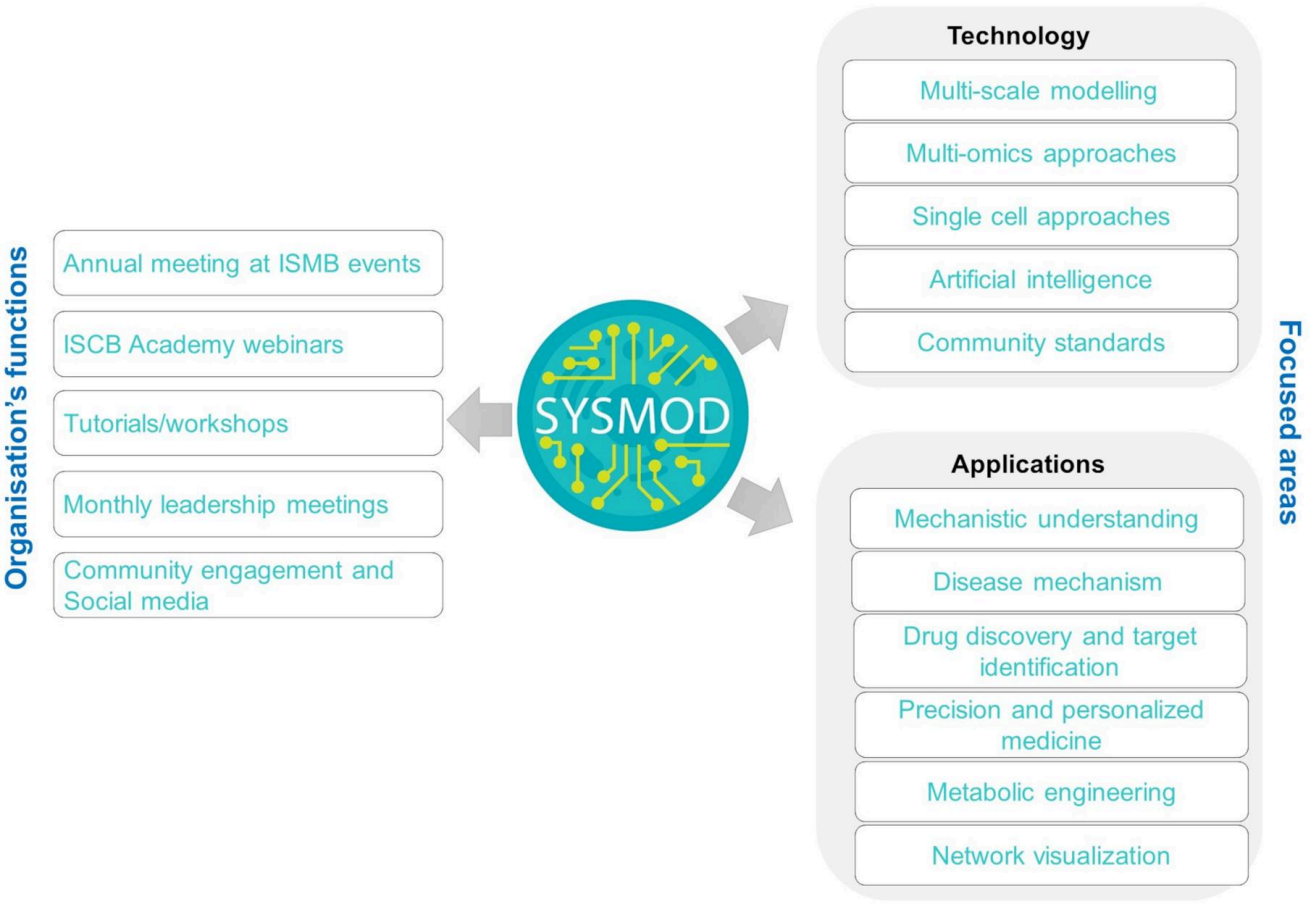
Overview of SysMod organization. SysMod COSI organizes an annual meeting at the ISMB conference and biannual ISCB academy webinars, tutorials, and workshops.

The SysMod COSI of ISCB serves as a platform for computational biologists, bioinformaticians, computer scientists, biologists, mathematicians, engineers, and others to share and discuss their knowledge and applications in systems biology and bioinformatics (Fig. 2). With eight annual conferences (in person and virtual), this forum has been a great platform to discuss the latest developments in mechanistic and predictive computational models of biological systems, analysis approaches, and applications in different areas, including human health, pharmaceuticals, the bioprocess industry, and agriculture. The community plays a crucial role in addressing the complexity of biological processes and networks, by employing numerous modeling approaches and integrating multi-omics data, clinical data, and AI approaches. These approaches develop dynamic, quantitative, or qualitative models of molecular processes such as metabolism, signalling, gene regulation, and cell–cell communication to organism–environment interactions. Such discussions are critical for enhancing the creation of more accurate, detailed, and reusable predictive models.

### 4.2 SysMod annual meeting and contributions

**Figure 3.**
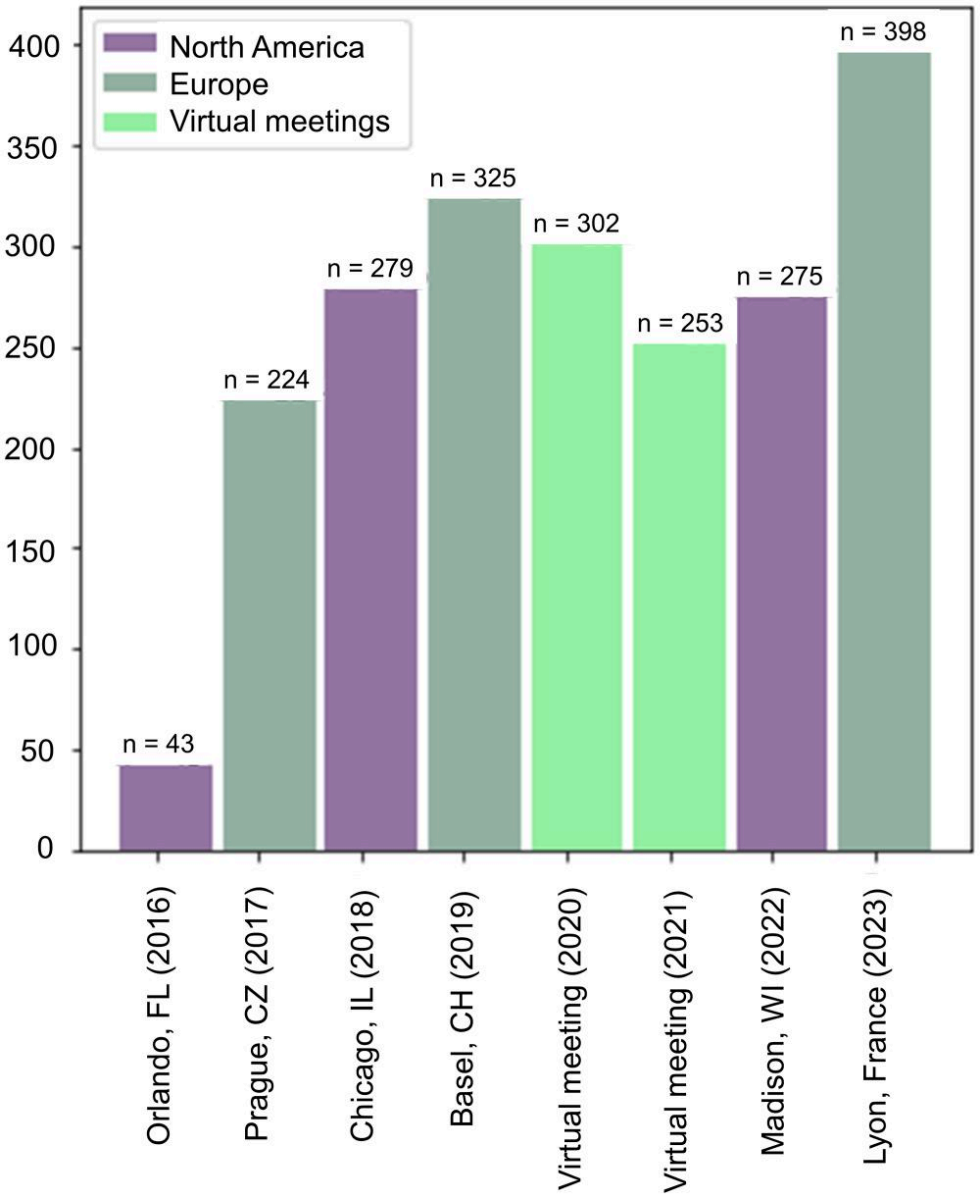
Participants in SysMod annual meeting across the globe. Numbers of registered participants at SysMod meetings (both in-person and virtual) from 2016 to 2023 across North America and Europe.

SysMod meetings have evolved into dynamic events hosted across North American and European subcontinents, attracting increasing numbers of participants (shown in Fig. 3), talks and poster abstract contributions, reflecting the growing interest in the computational systems modeling community. The SysMod meetings organized at the ISMB/ECCB conferences are one- to two-day events featuring various types of talks: keynote-themed, technical, lightning, and poster presentations from various career levels (post early and late) (Dräger *et al.*, 2021, Niarakis *et al.*, 2022, Puniya and Dräger, 2023). SysMod acknowledges outstanding research by awarding the best poster titles and financial assistance to early career researchers via merit-based travel awards. The community provides several online resources to promote engagement among its members and beyond. These include but are not limited to a SysMod-focused Google Group (sysmod@googlegroups.com), a website containing all the information about past and upcoming annual meetings (https://sysmod.info), scientific knowledge and event information dissemination via social media platforms. These include a YouTube channel (@sysmod) featuring recorded talks, lectures from previous years annual ISMB events, the biyearly ISCB webinars from the invited speakers, and tweets summarizing the talks at the annual conference via SysMod’s X account (@cosi_sysmod). These resources encourage collaboration and knowledge exchange among researchers from diverse fields, furthering the community’s efforts in understanding biological systems and their applications in biology, medicine, agriculture, and industry and enhancing the community outreach.

## 5 Future perspectives

The computational modeling field is poised for significant growth, driven by advancements in computational power and methodologies. The field is expected to advance in addressing the challenges of model scalability, generalizability, and applicability. We anticipate models becoming more comprehensive, integrating multi-omics data and spanning various biological scales. Incorporating ML and other AI techniques could enhance these model’s efficiency and predictive power. With growing data from single-cell technology, the vital area of future innovation will also include integrating spatial and temporal data into models, enhancing our understanding of dynamic biological systems. With biological data becoming increasingly complex, standardizing data formats and guidelines, such as FAIR (Findable, Accessible, Interoperable, and Reusable) data principles (Wilkinson *et al.*, 2016), and modeling protocols will become more critical. The community should focus on enhancing the model reproducibility and ensuring that models and findings are widely available to the research community.

## 6 Conclusion

Understanding complex biological systems requires multidisciplinary approaches. Insights from biology, computer science, mathematics, and other fields are helpful to address the common challenges in modeling complex biological systems. SysMod COSI brings together experts from various disciplines to address the pressing challenges of the field. For readers interested in contributing to this rapidly evolving field, engaging with SysMod COSI presents an excellent opportunity. With this, we encourage increased engagement and active participation in SysMod COSI and urge readers to contribute to ongoing efforts to enhance our understanding of complex biological systems and their applications.

## Data Availability

The data underlying this article are available in the article. The details about SysMod meetings can be found at https://sysmod.info/.
